# Costs of (In)visibility: Issues of Disclosure and Data for Health Equity Research with Sexual and Gender Minority Veterans

**DOI:** 10.1089/heq.2023.0005

**Published:** 2023-05-26

**Authors:** John R. Blosnich

**Affiliations:** ^1^ Center for Health Equity Research and Promotion, VA Pittsburgh Healthcare System, Pittsburgh, Pennsylvania, USA.; ^2^ Suzanne Dworak-Peck School of Social Work, University of Southern California, Los Angeles, California, USA.

**Keywords:** electronic health records, sexual orientation, gender identity, health equity

## Abstract

Sexual and gender minority (SGM) veterans experience numerous disparities in health conditions, behavioral risks, and social adversities compared with non-SGM veterans. Although survey data have illuminated these differences, SGM veterans remain largely invisible in administrative data such as electronic health records due to lack of sexual orientation and gender identity information. Administrative data hold promise for propelling SGM health equity research, but several issues must be addressed, including weighing the benefits and threats of visibility for SGM people in data sets that are tied to receipt of services.

## Introduction

Disparities among people who identify as sexual minority (e.g., lesbian, gay, and bisexual) and/or gender minority (e.g., transgender and nonbinary)—or sexual and gender minority (SGM)—are well documented, including disparities in health conditions (e.g., asthma and depression), health risk behaviors (e.g., substance use and suicide attempt), and adverse social determinants (e.g., housing instability, discrimination, violence victimization, and family rejection).^1^ The documentation of health disparities is often conceptualized in a framework of three research generations that first detect disparities among groups, then identify the mutable determinants producing those disparities, which ultimately informs intervention and prevention research to reduce those disparities.^2^

For SGM populations, progress along the generations framework has been vexed primarily by the limited availability of data sets that include sexual orientation and gender identity (SOGI) information. For example, only in 2014 did the Behavioral Risk Factor Surveillance System add an official, but optional, SOGI survey module, and the National Survey of Drug Use and Health added sexual orientation items in 2015.

These large surveys afforded opportunities to examine the intersection of SGM and veteran statuses.^3–5^ Datamining of Veterans Health Administration (VHA) electronic health records (EHR) also jumpstarted health disparities research about SGM veterans. Crucial as these efforts were for information about SGM veterans, they need to be appraised and contextualized within the datasphere to chart next steps for health equity research.

## Generations of Disparities Research for SGM Veterans

Over the past decade, studies of SGM veterans largely replicated disparities found in general population samples (see Kauth et al. for a review up through 2014^6^). For example, several studies of survey data documented greater alcohol and tobacco use as well as poorer mental health among SGM veterans than non-SGM veterans.^3–5,7–9^ Other studies showed greater adverse social determinants, including sexual minority veterans more likely to report discrimination than heterosexual veterans^8,10^ and transgender VHA veterans having nearly three times the prevalence of housing instability compared with cisgender VHA veterans.^11,12^

Several studies of SGM veterans through VHA EHR data suggest numerous disparities (e.g., post-traumatic stress disorder, heart disease, and eating disorders),^12,13^ including greater rates of suicidal ideation, attempt, and suicide mortality than non-SGM veterans.^14–16^

These first-generation studies were calls to action for a progression of research to identify the mutable mechanisms producing disparities and to evaluate interventions, programs, and policies for reducing disparities. Unfortunately, second- and third-generation research are scant, with some focusing on policy-level associations with health outcomes^17^ and access to care,^18^ others on the representation of SGM veterans' utilization of VA care programs,^19,20^ and only one tailored intervention tested with SGM veterans.^21^

## Persistent Data Challenges

In SGM health equity research, there are three related challenges around data collection: convincing stakeholders to include SOGI information in data collection systems, discerning how to collect SOGI information, and creating environments in which people—particularly SGM people—feel comfortable to disclose SOGI data. All the work to surmount the first two challenges can be for naught if SGM people do not feel safe to disclose their information. Inclusion of SOGI in self-reported survey data projects has been underway and continues to expand, bolstered by the typically anonymous or confidential nature of survey research and its low stakes: surveys typically are not tied to anything of consequence for the participant.

Conversely, inclusion of SOGI data in administrative data, such as EHR, has different stakes because disclosure becomes part of identifiable records linked to some current or future service. These stakes can be quite high for SGM individuals for several reasons including fear of discrimination in care.^22^ Fears of discrimination loom largely for many veterans because of the legacy of the department of defense's “Don't Ask, Don't Tell” policy, which barred sexual minorities from serving openly and led to discharges of active-duty personnel. Even years after the repeal of “Don't Ask, Don't Tell,” SGM veterans discuss concerns rooted in its legacy, including misinterpretation of it as a VA policy and fears that SOGI information in VHA could jeopardize their military benefits.^23^

The current absence of sexual orientation data and limited gender identity data in VHA EHR has forced researchers to rely on proxy methods to identify SGM populations. For research about transgender and gender diverse veterans, this has meant reliance on International Classification of Disease (ICD) codes related to gender identity disorder (GID). Importantly, the ICD system still uses GID, whereas the *Diagnostic and Statistical Manual, 5th Revision* (DSM-5) replaced GID with gender dysphoria. Although using ICD codes identifies a subpopulation of transgender and gender diverse veterans (i.e., those necessitating an ICD diagnosis),^24^ not all transgender and gender diverse veterans need a diagnosis code.

Moreover, diagnostic coding pathologizes gender identity. For sexual minority veterans, lack of structured data such as ICD codes has necessitated complex methods of natural language processing and machine learning to extract sexual orientation information from unstructured data (i.e., clinical notes written by providers).^15^

Although these data techniques supported research highlighting the needs of SGM veterans, they are not ideal for identifying the population of interest due to several limitations (e.g., provider documentation biases and clerical or coding errors). More importantly, these methods rarely can produce an adequate comparison of heterosexual and cisgender veterans because heterosexuality and cisgender identities are rarely documented in EHR in the ways that minoritized identities are. Consequently, the *lack* of SGM-related terms or codes is often interpreted as patients being cisgender or heterosexual, which creates potential misclassification bias.

Aside from these analytic critiques of using proxy measures to operationalize SOGI, the methods also magnify an injustice in data. Imagine if race and ethnicity data were unavailable in EHR, and health equity researchers had to use methods to identify proxies for patients' racial and ethnicity identities in clinical notes. Would we trust that providers would note such information explicitly? Would we justify the use of ICD codes that we thought might be more prevalent among individuals with minoritized racial and ethnic identities?

Such studies would likely—and rightfully—be met with intense scrutiny. Yet, for health equity research with SGM veterans, it suffices as “the best we could do” and “better than nothing.” When it comes to administrative data systems and SGM populations, it is long past time to hang up “better than nothing” and demand better.

## Forging the Administrative Data Frontier

For several reasons, the absence of SOGI in administrative data systems is a problem that health equity researchers must tackle alongside SGM communities. First, administrative data can facilitate more real-time evaluation of the reach and utilization of services than what is afforded by costly time-intensive survey projects. For example, Montgomery and colleagues used VHA administrative data to discover that transgender and gender diverse veterans were more likely to utilize several kinds of permanent supportive housing programs compared with cisgender veterans;^19^ a positive finding, given that transgender and gender diverse veterans are more likely than cisgender veterans to report housing instability.

With evidence of several other disparities, VHA administrative data could determine SGM veterans' utilization of care and services designed to address those disparities. For example, SGM veterans are more likely to smoke than non-SGM ceterans,^5,7,12^ but are SGM veterans also more likely to receive evidence-based smoking cessation treatments from VHA compared with their non-SGM veteran peers?

Second, administrative data can fill gaps in crucial knowledge about outcomes that, again, would take years to determine through survey data. For instance, SOGI data are not included in U.S. mortality surveillance,^25^ but by utilizing VHA's Mortality Data Repository, researchers have provided inaugural findings about mortality among SGM veterans, revealing starkly higher rates of suicide than the general VHA population.^15,16^

Lastly, administrative data provide the benchmarks against which prevention and intervention research show effectiveness. In the example of mortality data, if an intervention effort aimed to prevent suicide, at scale, in SGM populations, it would be impossible to evaluate without data on suicide deaths for SGM people. It seems tautological, but it is the reality for much of SGM research pursuant to that third-generation research to ameliorate health disparities: how can one reduce a rate without having said rate?

It is incumbent on health systems and providers to clearly communicate the purposes of including SOGI data. A common reluctance from both patients and providers is whether SOGI data are relevant to health care provision. In an ideal world where biases—both within and external to health care systems—would not exist, perhaps patient demographics would not matter for health care. An analogue would be questioning the need of racial and ethnic identity data if health care systems strive to be “colorblind.” This argument fails because society is not colorblind. Because biases operate overtly and covertly, systems must have data to be held accountable for detecting not *if* inequities exist in their operations, but *the extent of* inequities, whether they be in access, utilization, or outcomes.

The importance of including SOGI measures in administrative data will need to address concerns around safety to disclose, information security, and flexible data systems to accommodate the developmental nature of SGM identities. Balancing costs of visibility (e.g., discrimination) and invisibility (e.g., erasure) for SGM veterans requires conversations at all levels of health care, but they must be centered on patient and community voices.

## Author's Contribution

J.R.B. was solely responsible for the conceptualization, composition, and reviewing of all drafts.

## Abbreviations Used

DSM-5: *Diagnostic and Statistical Manual, 5th Revision*
EHRs: electronic health records
GID: gender identity disorder
ICD: International Classification of Disease
SGM: sexual and gender minority
SOGI: sexual orientation and gender identity
VHA: Veterans Health Administration
